# Major Histocompatibility Complex Based Resistance to a Common Bacterial Pathogen of Amphibians

**DOI:** 10.1371/journal.pone.0002692

**Published:** 2008-07-16

**Authors:** Seth M. Barribeau, Jandouwe Villinger, Bruce Waldman

**Affiliations:** School of Biological Sciences, University of Canterbury, Christchurch, New Zealand; University of Uppsala, Sweden

## Abstract

Given their well-developed systems of innate and adaptive immunity, global population declines of amphibians are particularly perplexing. To investigate the role of the major histocompatibilty complex (MHC) in conferring pathogen resistance, we challenged *Xenopus laevis* tadpoles bearing different combinations of four MHC haplotypes (*f*, *g*, *j*, and *r*) with the bacterial pathogen *Aeromonas hydrophila* in two experiments. In the first, we exposed *ff*, *fg*, *gg*, *gj*, and *jj* tadpoles, obtained from breeding MHC homozygous parents, to one of three doses of *A. hydrophila* or heat-killed bacteria as a control. In the second, we exposed *ff*, *fg*, *fr*, *gg*, *rg*, and *rr* tadpoles, obtained from breeding MHC heterozygous parents and subsequently genotyped by PCR, to *A. hydrophila*, heat-killed bacteria or media alone as controls. We thereby determined whether the same patterns of MHC resistance emerged within as among families, independent of non-MHC heritable differences. Tadpoles with *r* or *g* MHC haplotypes were more likely to die than were those with *f* or *j* haplotypes. Growth rates varied among MHC types, independent of exposure dose. Heterozygous individuals with both susceptible and resistant haplotypes were intermediate to either homozygous genotype in both size and survival. The effect of the MHC on growth and survival was consistent between experiments and across families. MHC alleles differentially confer resistance to, or tolerance of, the bacterial pathogen, which affects tadpoles' growth and survival.

## Introduction

The major histocompatibility complex (MHC) encodes cellular mechanisms that determine immunological self/non-self recognition in vertebrates. Genetic relatives share MHC alleles, which encode T-cell repertoires, so their immune systems should recognize similar arrays of pathogens. Because MHC alleles are codominant, individuals that are heterozygous at the MHC should have a larger immunological repertoire than homozygotes [1]. This fitness advantage may accumulate over a lifetime. While particular MHC-homozygous genotypes may confer resistance to certain pathogens, MHC-heterozygous genotypes might cope better with sequential or simultaneous infections by different pathogens [2], [3].

Unlike those of many other vertebrates, African clawed frog (*Xenopus laevis*) MHC class I and II loci are tightly linked [4], [5], which facilitates studies of genetic determinants of immune responses. *Xenopus* tadpoles express MHC class I molecules only in the epithelial tissue of some organs such as gills, lungs, and intestine [6], and class II molecules on B cells and antigen-presenting cells [7]. Despite their limited MHC expression, tadpoles are immunocompetent, although they are more susceptible than adults to viral infections [8].

Amphibian populations have been declining worldwide, and pathogens may be responsible for many population declines [9]–[14]. The role of the MHC in conferring disease resistance in amphibians has received only limited study despite its obvious importance for vertebrate conservation programs [15], [16]. Gantress *et al.* [8] found that inbred *X. laevis* with particular MHC haplotypes were more susceptible than others to the ranavirus frog virus-3. Amphibian population declines, however, have been linked to a number of pathogens, including the amphibian chytrid fungus *Batrachochytrium dendrobatidis* [12], [13], [17]–[20], iridoviruses [21], [22], and the bacterium *Aeromonas hydrophila* [23]–[27]. Of these, *A. hydrophila* is considered a secondary pathogen [21], [28], [29] that is likely to infect immunocompromised animals [30], [31]. Clearly, designing effective management strategies requires some understanding of amphibian immune responses to a diverse range of pathogens.

We examined whether MHC genotype affected the survival and growth of *X. laevis* tadpoles challenged with *A. hydrophila*. Tadpole growth rates predict size [32]–[34], timing [32], [35] and survival [33], [36] to metamorphosis, and size [37], time and survival to first reproduction [37], [38], all measures of fitness. Even if tadpoles survive, reduced growth might indicate sub-lethal effects of pathogen exposure [39]. We exposed tadpoles that bore diploid combinations of four different MHC haplotypes to inocula of *A. hydrophila*. First, we examined the effects of pathogen exposure on tadpoles with different MHC genotypes across several families. We then compared the resistances of MHC genotypes within families. This allowed us to assess whether the same patterns of MHC resistance emerged within as among families, independent of non-MHC heritable differences.

## Materials and Methods

### (a) Biological materials

#### (i) Animals

We bred *Xenopus laevis* frogs with known sequences for MHC class I and class II alleles. The haplotypes are designated *f*, *g*, *j*, and *r* (GenBank class Ia accession numbers: AF185579, AF185580, AF185582, AF185586 [40]; class II accession numbers: AF454374–AF454382). These frog strains originated from the Basel Institute for Immunology.

Between 13:00 and 15:00 on the day of breeding, we isolated and primed females by injecting their dorsal lymph sac with 0.03 mg luteinizing hormone–releasing hormone (LH-RH; Argent Chemical Laboratories, Redmond, Washington, USA) dissolved in 150 µL of autoclaved double distilled water. We monitored the cloacae of the frogs from 5 to 8 h after priming. Once cloacae displayed swelling and redness due to increased blood flow, we injected the females with an additional 0.1 mg LH-RH dissolved in 500 µL of autoclaved double distilled water, and immediately placed them into breeding tanks. To ensure that the breeding pair would not consume the eggs, we covered the substrate of breeding tanks with a plastic mesh grid which allowed fertilized eggs to fall through to the bottom. For breeding frogs and rearing tadpoles, we used aerated, carbon-filtered Christchurch city municipal water, which is sourced from deep-water aquifers without chemical treatment.

#### (ii) Bacteria

We isolated a strain of *Aeromonas hydrophila* from the heart of an adult *X. laevis* that died at our facility in January 2003. Subcultures of the original heart culture were maintained at −80°C in CryoBeads (Pro-Lab Diagnostics, Wirral, UK). All bacteria used for exposures were descended through no more than three generations from the original isolation. We cultured these bacteria on tryptone soya agar (TSA; Oxoid, Basingstoke, UK) and incubated the plates aerobically for 24 h at 32°C. We introduced a single colony into a universal bottle of tryptone soya broth (TSB; Oxoid, Basingstoke, UK) and incubated it aerobically for 24 h at 32°C. After incubation, we transferred 10 mL of the broth culture into 1 L of TSB in Erlin-Meyer flasks. We incubated these flasks aerobically for 24 h at 32°C shaking at 200 rpm. We quantified the cultures by triplicate serial dilution plate counts the day before experimental exposure.

### (b) Experiment 1. Does resistance to *A. hydrophila* vary by MHC genotype?

#### (i) Subjects

We bred 3 male and 3 female MHC-homozygous (*ff*, *gg*, *jj*) *X. laevis* frogs, each sequentially with two partners, during one night (Table 1). We paired MHC-identical homozygotes first, and after they began spawning, we separated the pairs and allowed them to continue mating with partners whose MHC genotype differed from their own. Later that night, we repeated this procedure to control for egg order effects by creating early and late clutches of each MHC genotype. This produced tadpoles with 6 genotypes (*ff*, *fg*, *fj*, *gg*, *gj*, *jj*) from 12 clutches of eggs. MHC heterozygous tadpoles were half-siblings of the MHC homozygous tadpoles (i.e. *fg* tadpoles are half-siblings of *ff* and *gg* tadpoles) to limit non-MHC heritable differences. Two days after hatching, we placed 100 tadpoles from each clutch into separate 10 L high-density polyethylene tanks.

**Table 1:** Among-families experimental design; sample sizes by genotype and treatment.

Brood	Parental MHC types		Tadpole MHC type	Exposure (cfu/ml)			Control (heat-killed bacteria)
	♀	♂		1.0×10^6^	2.5×10^6^	3.0×10^6^	
1 (early)	*ff*	*ff*	*ff*	8	8	8	8
	*gg*	*gg*	*gg*	8	8	8	8
	*jj*	*jj*	*jj*	8	8	8	8
2 (early)	*ff*	*gg*	*fg*	8	8	8	8
	*gg*	*jj*	*gj*	8	8	8	8
	*jj*	*ff*	*fj*	8	8	8	8
3 (late)	*ff*	*ff*	*ff*	8	8	8	8
	*gg*	*gg*	*gg*	8	8	8	8
	*jj*	*jj*	*jj*	8	8	8	8
4 (late)	*ff*	*gg*	*fg*	8	8	8	8
	*gg*	*jj*	*gj*	8	8	8	8
	*jj*	*ff*	*fj*	8	8	8	8

#### (ii) Procedures

To obtain baseline size measurements of the tadpoles prior to inoculating them with bacteria, we randomly selected 32 tadpoles from each clutch, 16 days after hatching. We photographed each in its own Petri dish from 60 cm directly above with a Nikon Coolpix 4500 digital camera. A 10 cm ruler was included in the photographs for scale. We measured body length (BL, from the tip of the snout to the vent at the base of the tail) and total length (TL, from the tip of the head to the tip of the tail) from digital images using NIH ImageJ 1.3 (National Institutes of Health, Bethesda, Maryland, USA). We then placed each tadpole into an individual 1 L polypropylene beaker (day 0).

We exposed tadpoles by pipetting an inoculum of *A. hydrophila* (1.0×10^6^ colony forming units/ml (cfu/ml), 2.5×10^6^ cfu/ml, 3.0×10^6^ cfu/ml) into the water in their beaker (Table 1). These doses are less than or equal to *A. hydrophila* concentrations in nature [41]. Control tadpoles were inoculated with 3.0×10^6^ cfu/ml of *A. hydrophila*, killed by autoclave at 121°C, 103 kPa for 20 min. Each treatment comprised 8 tadpoles. We moved each beaker one place every day, within two-replicate blocks (48 beakers in a 12×4 grid), to control for position effects. Tadpoles were fed every second day with ground nettle suspension and the water was topped up to 1 L every 4 days to compensate for evaporation. Ten, 25, and 35 days after exposure, we photographed and measured the tadpoles as before.

We first compared Kaplan-Meier survival curves with log rank tests using the survdiff procedure in R 2.3.0 (R Development Core Team, Vienna, Austria). The survival curves allow inspection of gross differences in survival over time. We then analyzed how total mortality at day 35 was affected by MHC genotype, bacterial dose, clutch (early and late), and block with a generalized linear mixed model (GLMM) using the glmmML package in R (Göran Broström, Department of Statistics, Umeå University) with binomial error distribution and logit-link function. The glmmML package fits models using maximum likelihood estimation. We treated genotype, bacterial dose, clutch and block as fixed variables, and subject (individual identity) as a random variable. We included starting body length as a covariate. We compared body and total lengths associated with the same fixed factors using repeated-measures ANOVA. We compared the lengths of control tadpoles to those of tadpoles exposed to bacteria by orthogonal contrasts. All repeated measures analyses were conducted with Statistica 6.1 (Statsoft, Tulsa, Oklahoma, USA) using Type III sums of squares.

Despite using half-siblings to limit the heritable effects of non-MHC genes, these genes still may have had effects on disease resistance. To control for non-MHC variation, we conducted within-family tests in an additional experiment, as follows.

### (c) Experiment 2. Does susceptibility to disease correspond to MHC genotype within families?

#### (i) Subjects

We crossed three pairs of MHC-identical heterozygous frogs (*fg*×*fg*, *fr*×*fr*, *rg*×*rg*) to produce clutches of mixed homozygotes and heterozygotes (e.g., *rr*, *rg*, *gg*; Table 2). Insufficient numbers of frogs heterozygous with the *j* haplotype were available for us to include in these analyses. The following day, we removed 200 eggs from each clutch and placed them individually into 1 L polypropylene beakers.

**Table 2:** Within-families experimental design; sample sizes by genotype and treatment.

Cross	Genotype	Exposed	Control (heat-killed bacteria)	Control (clean media)
*fg*×*fg*	*ff*	10	10	-
	*fg*	10	10	11
	*gg*	10	10	-
*fr*×*fr*	*ff*	14	14	-
	*fr*	16	16	16
	*rr*	16	16	-
*rg*×*rg*	*rr*	15	15	-
	*rg*	16	16	16
	*gg*	13	13	-

#### (ii) Procedures

Two weeks after hatching, we genotyped 150 tadpoles from each clutch for MHC type [42]. Three weeks after hatching, we photographed and measured the tadpoles, as before, and exposed them to *A. hydrophila*, heat-killed *A. hydrophila*, or pelleted clean bacterial media by pipetting the inocula into the tadpoles' water. The pelleted clean bacterial media served as a second control. We exposed the tadpoles to an initial bacterial dose of 2.0×10^7^ cfu/ml, the same dose of heat-killed bacteria, or a pellet from the same volume of clean media.

The numbers of each genotype that were produced in the spawn limited the sample sizes (Table 2). We arranged the beakers in single-family blocks with 5 beakers across each block. Tadpoles in each row of beakers were of the same genotype and in the same treatment group. We moved each row one position every day within the family blocks to ensure that all tadpoles were exposed to the same position effects.

The initial dose of bacteria failed to induce mortality so we increased the exposure dose. On day 5, we exposed the tadpoles to 4.0×10^7^ cfu/ml of *A. hydrophila*. On day 18, we photographed and measured all tadpoles, and cut a small (<3 mm) section of tail to create a portal for the bacteria. We then re-photographed the tadpoles, and exposed them to 6.0×10^7^ cfu/ml of *A. hydrophila*. We photographed and measured the tadpoles for the final time on day 28.

We compared Kaplan-Meier survival curves with log rank tests using the survdiff procedure as before. We analyzed how mortality, at the end of the experiment (day 28), was affected by MHC genotype, family nested within genotype, and bacterial exposure using a generalized linear mixed model (GLMM, R 2.3.0) with binomial error distribution and logit-link function. Family, corresponding to the breeding regimen (*fg*×*fg*, *fr*×*fr*, *rg*×*rg*), was nested within genotype to examine whether the effect of genotype was consistent across families despite heritable non-MHC differences. We included tadpole identity in the model as a random factor, and starting body size as a covariate. We compared the lengths of tadpoles in the two control groups separately with repeated-measures ANOVA. We used tadpoles exposed to heat-killed bacteria as controls in all other analyses. We analyzed length data by repeated-measures ANOVA with exposure and MHC genotype, and family nested within genotype, as fully factorial main effects using Type III sums of squares (Statistica 6.1). To establish whether tadpoles differed in size between the two experiments, we compared the length of tadpoles at day 0 between experiments with a two-sample t test.

## Results

### (a) Experiment 1. Does resistance to *A. hydrophila* vary by MHC genotype?

#### (i) Mortality

**Figure 1:**
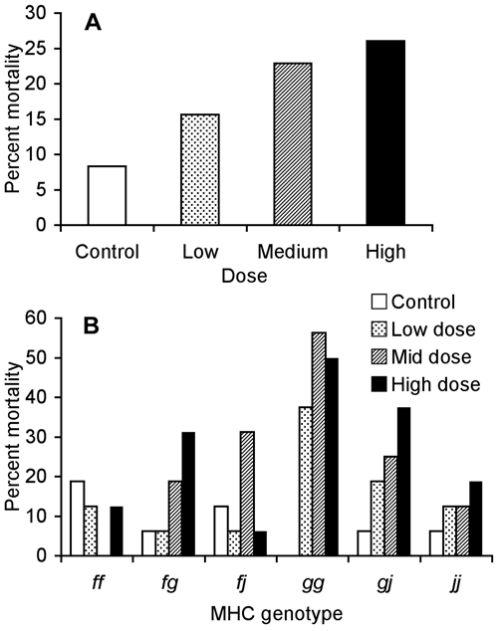
Mortality as a function of bacterial dose and MHC genotype among families. (A) Percent mortality of tadpoles exposed to the control (3.0×10^6^ cfu/ml heat-killed), low (1.0×10^6^ cfu/ml), medium (2.5×10^6^ cfu/ml), and high (3.0×10^6^ cfu/ml) doses of *A. hydrophila*. N = 90 in each treatment. (B) Percent mortality of tadpoles from each MHC genotype that were exposed to each dose of live *A. hydrophila* or the control. N = 15 in each condition.

**Figure 2:**
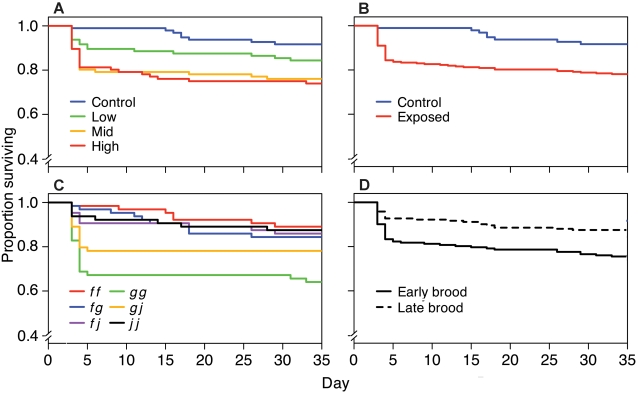
Survival with time as a function of bacterial dose, MHC genotype, and clutch order among families. Kaplan-Meier plots showing the survival of (A) tadpoles exposed to the control (3.0×10^6^ cfu/ml heat-killed), low (1.0×10^6^ cfu/ml), medium (2.5×10^6^ cfu/ml), and high (3.0×10^6^ cfu/ml) doses of *A. hydrophila*; (B) tadpoles exposed to the control or *A. hydrophila* (all doses combined); (C) tadpoles from each MHC genotype; and (D) tadpoles from early and late clutches.

Tadpole mortality was affected by exposure to *A. hydrophila*, the tadpoles' MHC genotype, and clutch order. Tadpoles exposed to higher doses of *A. hydrophila* suffered more mortality (Fig. 1a; *F*_3,328_ = 4.88, *P* = 0.0025). Tadpoles exposed to the high and intermediate doses died before tadpoles exposed to the low dose and control tadpoles, although the dose survival curves did not differ significantly (Fig. 2a; *χ^2^* = 5.8, 3 d.f., *P* = 0.12). Exposed tadpoles died sooner than control tadpoles (Fig. 2b; *χ^2^* = 3.8 , 1 d.f., *P* = 0.05). Most mortality occurred within 5 days of pathogen exposure.

Some MHC genotypes suffered less mortality than others (Fig. 1b; *F*_5,328_ = 4.30, *P* = 0.0008). Survival curves also differed among MHC genotypes (Fig. 2c) although this result only approached significance (*χ^2^* = 10.0, 5 d.f., *P* = 0.074). Furthermore, the influence of exposure dose on mortality differed with MHC genotype (*F*_15,328_ = 1.91, *P* = 0.022). Certain MHC genotypes appear especially susceptible to *A. hydrophila*; *gg* tadpoles had the highest rate of mortality when exposed to the bacterium (43%) but none of the *gg* control tadpoles died. In contrast, *ff* tadpoles did not suffer increased mortality when exposed to the pathogen. The *fg* tadpoles had mortality rates intermediate to their *ff* and *gg* half-siblings (Fig. 1b). A similar pattern is apparent in the *gg*, *gj*, and *jj* tadpoles.

Tadpoles from earlier clutches were more likely to die (8.9%) than their full siblings from later clutches (3.4%; *F*_1,328_ = 10.60, *P* = 0.0013) but the survival curves did not differ (Fig. 2d; *χ^2^* = 0, 1 d.f., *P* = 0.934). Initial body length did not affect mortality (survivors' initial BL: 5.20±0.03 mm, dead tadpoles' initial BL: 5.27±0.07 mm, *X̅*±SE, *F*_1,328_ = 0.21, *P* = 0.65).

#### (ii) Length

**Figure 3:**
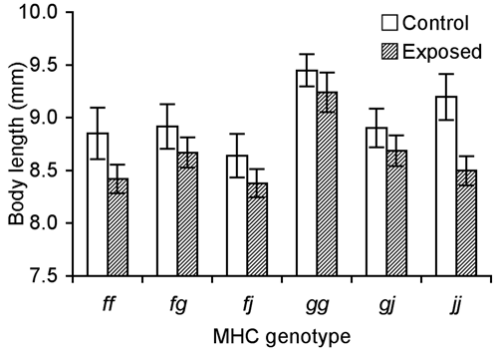
Growth as a function of MHC genotype among families. Body length (*X̅*±SE) at day 25 of tadpoles from each genotype exposed to the pathogen *A. hydrophila* and the control.

Tadpoles significantly differed in length as a function of their MHC genotype (Fig. 3; BL: *F*_5,258_ = 6.50, *P*<0.001; TL: *F*_5,258_ = 8.26, *P*<0.001). The largest and smallest tadpoles were of *gg* and *fj* genotypes respectively. This trend remained consistent (day 0, *gg* BL: 5.64±0.06 mm, TL: 13.84±0.14 mm; *fj* BL: 5.11±0.07 mm, TL: 13.11±0.16 mm; day 34, *gg* BL: 12.04±0.39 mm, TL: 32.2±0.64 mm; *fj* BL: 11.57±0.19 mm, TL: 31.07±0.54 mm). Tadpoles with MHC genotypes that suffered lower mortality (*f* and *j* haplotypes; Fig. 1b) tended to grow less when exposed to the pathogen than did those with susceptible genotypes (haplotype *g*; Fig. 3). However, responses of MHC genotypes did not differ in response to exposure dose (BL: *F*_15,258_ = 0.46, *P* = 0.96; TL: *F*_15,258_ = 0.78, *P* = 0.70).

Tadpoles that developed from clutches laid earlier in the evening were significantly smaller than those laid later in the same evening. On day 25, tadpoles from the later clutches were 4% larger than tadpoles from earlier clutches (BL: early 8.61±0.07 mm, late 8.81±0.07 mm, *F*_1,258_ = 4.60, *P* = 0.033; TL: early 21.07±0.28 mm, late 21.92±0.25 mm, *F*_1,258_ = 7.10, *P* = 0.008), but by day 34 there was no difference in size between tadpoles of the two clutches (BL: early 11.73±0.11 mm, late 11.80±0.09 mm, *F*_1,258_ = 0.43, *P* = 0.51; TL: early 31.18±0.34 mm, late 31.43±0.29 mm, *F*_1,258_ = 0.59, *P* = 0.44).

Control tadpoles were significantly larger (TL: 22.36±0.34 mm) than all those exposed (21.19±0.22 mm) at day 25 (BL: *F*_1,258_ = 15.42, *P* = 0.0001; TL: *F*_1,258_ = 12.75, *P* = 0.0043). Thirty-four days after exposure, surviving tadpoles that were exposed to the pathogen were of similar size to the control tadpoles (BL: control 11.78±0.13 mm, exposed 11.76±0.09 mm, *F*_1,258_ = 0.0051, *P* = 0.94; TL: control 31.21±0.42 mm, exposed 31.40±0.27 mm, *F*_1,258_ = 0.108, *P* = 0.74).

### (b) Experiment 2. Does susceptibility to disease correspond to MHC genotype within families?

#### (i) Mortality

**Figure 4:**
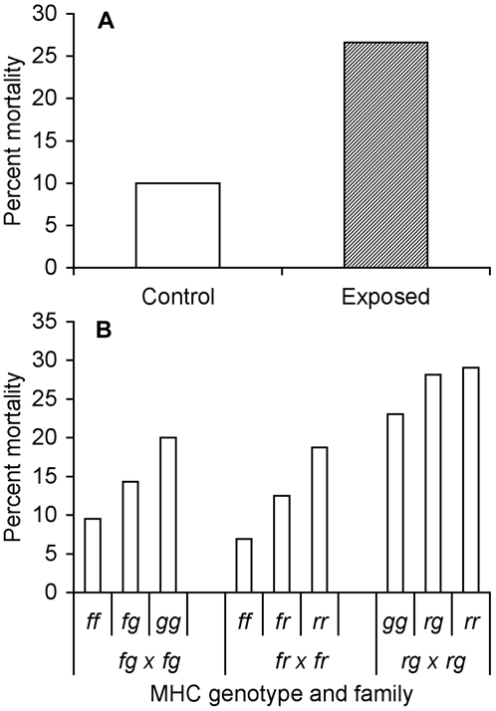
Mortality as a function of bacterial exposure and MHC genotype within families. (A) Percent mortality of tadpoles exposed to live (exposed) and heat-killed (control) *A. hydrophila*. N = 120 for each treatment. (B) Percent mortality of tadpoles with each MHC genotype from 3 different families. Sample sizes differed among families; see Table 1.

**Figure 5:**
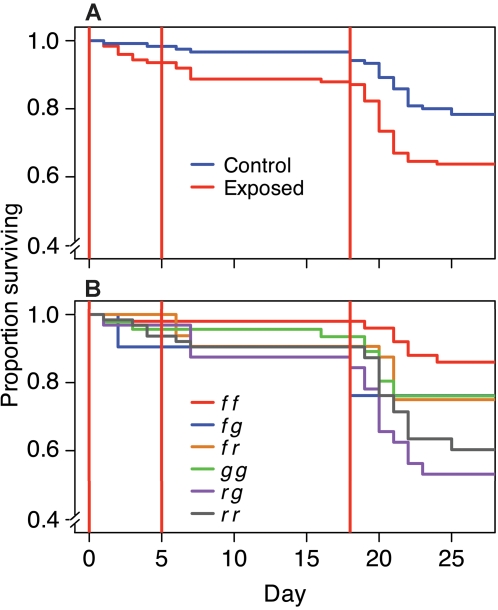
Survival with time as a function of bacterial exposure and MHC genotype within families. Kaplan-Meier plots showing the survival of (A) tadpoles exposed to live (exposed) or heat-killed (control) *A. hydrophila*, and (B) tadpoles with different MHC genotypes. Vertical lines indicate exposure days.

Tadpoles died in higher numbers when exposed to live rather than heat-killed *A. hydrophila* (Fig. 4a; *F*_1,228_ = 6.36, *P* = 0.012), and died sooner than controls (Fig. 5a; *χ^2^* = 6.4, 1 d.f., *P* = 0.011). MHC type significantly affected mortality (Fig. 4b; *F*_5,228_ = 4.71, *P* = 0.0004) and survival curves (Fig. 5b; *χ^2^* = 16.4, 5 d.f., *P* = 0.0057). Mortality did not differ among tadpoles of the same MHC genotypes from different families (*F*_3,228_ = 0.33, *P* = 0.80). More *rr* than *gg* tadpoles died, but both these MHC genotypes had higher mortality rates than *ff* tadpoles, which were relatively resistant to *A. hydrophila*. In each family, heterozygote mortality was intermediate to the two MHC homozygous genotypes (Fig. 4b). Exposure and MHC genotype showed no interaction in their effects on mortality (*F*_5,228_ = 0.48, *P* = 0.79). Tadpoles that were initially smaller were more likely to die during the course of the experiment than larger tadpoles (surviving tadpoles' initial BL: 6.22±0.06 mm, dying tadpoles' initial BL, 5.53±0.12 mm, *F*_1,228_ = 25.44, *P*<0.0001).

#### (ii) Length

**Figure 6:**
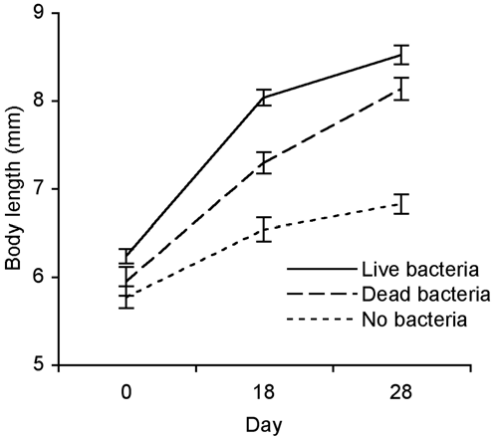
Growth as a function of bacterial exposure. Body length (*X̅*±SE) of tadpoles exposed to live *A. hydrophila*, heat-killed bacteria and no bacteria (controls) over time.

**Figure 7:**
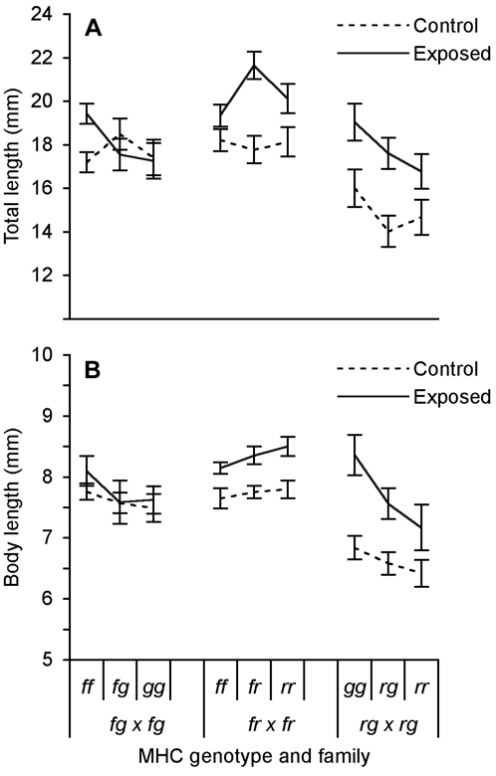
Growth as a function of MHC genotype within families. (A) Total and (B) body length (*X̅*±SE) of tadpoles on day 18 with different MHC genotypes that were either exposed to live or heat-killed *A. hydrophila* as a control.

Surviving tadpoles that had been exposed to live *A. hydrophila* were significantly larger than tadpoles that had been exposed to heat-killed bacteria (Fig. 6; BL: *F*_1,162_ = 11.02, *P* = 0.0011, TL: *F*_1,162_ = 9.71, *P* = 0.0022). Growth rates varied by MHC genotype (Fig. 7; BL: *F*_5,162_ = 3.79, *P* = 0.0090, TL: *F*_5,162_ = 2.66, *P* = 0.024). Among families, body length, but not total length, varied among individuals bearing the same MHC genotype (Fig. 7; BL: *F*_3,162_ = 5.86, *P* = 0.0026, TL: *F*_3,162_ = 0.63 *P* = 0.60). Overall, tadpoles were larger (BL 6.02±0.06 mm) in this experiment than those in the previous experiment (BL 5.22±0.03 mm; *t* = 12.63, 626 d.f., *P*<0.0001). Tadpoles that were exposed to heat-killed *A. hydrophila* were larger than those exposed to pelleted clean media (Fig. 6; BL: *F*_1,60_ = 19.88, *P*<0.0001, TL: *F*_1,60_ = 8.00, *P* = 0.0063).

## Discussion

We have shown (1) that exposure of *X. laevis* tadpoles to *A. hydrophila* affects their growth and survival, and (2) that the MHC mediates these responses. The effects of MHC genotype and bacterial exposure on survival were consistent–but effects on growth varied–between experiments. Tadpoles with the *r* or *g* haplotypes were susceptible to this pathogen and those with the *f* or *j* haplotypes were resistant to it. Heterozygous tadpoles with both susceptible and resistant haplotypes were always intermediate to either homozygote in both their growth and survival. This complements the previous finding that the *f* haplotype confers resistance and the *j* haplotype causes susceptibility to frog virus-3 [8]. The role of MHC genotype in conferring disease resistance is further suggested by the interaction that we found between MHC genotype and exposure on mortality.

In both experiments, *ff* tadpoles experienced low mortality, *gg* tadpoles suffered high mortality, and *fg* heterozygotes were intermediate to the two homozygous types. In the second experiment, *rr* homozygous tadpoles also suffered high mortality. The consistency of results from different genetic backgrounds suggests that the MHC, rather than other genes, determined bacterial resistance.

Differences in resistance conferred by MHC alleles have been documented in many vertebrates including fishes [43]–[47], mice [2], [48], birds [49], [50] and humans [51]–[53]. Most commonly, and as seen here, animals that are heterozygous at the MHC show disease resistance intermediate to the two homozygous genotypes [3], but over a lifetime of sequential infections with different pathogens or during co-infection, heterozygotes may benefit by having resistance superior to either homozygous genotype [2]. Individuals with common MHC haplotypes may be more susceptible to pathogens that evolve to avoid their defenses, thereby increasing the relative fitness of rare haplotypes [44], [47]. Both processes can drive MHC diversity and support the increased fitness of MHC heterozygotes in a dynamic environment (for reviews see [54]–[56]).

Immune responses protect individuals against pathogens and parasites, but can incur fitness costs [57], [58]. In the first experiment, tadpoles with resistant haplotypes (*f* and *j*), but not those with a susceptible haplotype (*g*), showed a trend of reduced growth when exposed to the pathogen, which suggests a possible tradeoff between growth and immune function. Tadpoles that grow faster or metamorphose at a larger size often accrue strong fitness advantages [32]–[38], so while certain MHC alleles may confer resistance to particular pathogens, individuals that bear them may have lower reproductive success. In the absence of the pathogen, selection may favor individuals bearing susceptible MHC alleles. Accelerating growth and development may represent a compensatory response of these individuals to the pathogen, as adults have stronger immune defenses than tadpoles [7], [8], [59]. Accelerated development in response to desiccation is accompanied by weaker cellular immune responses to antigens in wood frog (*Rana sylvatica*) tadpoles [57], possibly illustrating a tradeoff similar to that seen here.

Unlike in the first experiment, however, almost all genotypes in the second experiment grew more rapidly when exposed to the pathogen. This difference in response to the bacterial challenge might be due to our isolation of subjects into beakers earlier in the second experiment, which was necessary to genotype individuals. Consequently, subjects' growth, and probably their development, accelerated to a point at which they may have been less susceptible to the pathogen [7], [8], [60]. Indeed, a greater exposure dose was required to induce mortality, and smaller tadpoles were more likely to die than larger tadpoles.

Although exposed tadpoles in the second experiment grew larger, resistant tadpoles in the first experiment appear to have allocated less of their energy resources toward growth than did susceptible tadpoles. MHC class II molecules initiate immune responses to extracellular pathogens such as bacteria, and these class II molecules are expressed in high concentrations in the intestines of *X. laevis* [4]. Because *Xenopus* tadpoles are non-specific filter feeders, they ingest many species of bacteria and other microbial pathogens, potentially at high doses. The expression of MHC class II in the intestine may help these tadpoles respond to potentially dangerous microbial food. Thus, we would have expected tadpoles with greater resistance to *A. hydrophila* to utilize a potentially dangerous food source better than their siblings that have a weaker resistance. Nonetheless, tadpoles may have been selected to respond to immune stressors by reducing rather than increasing their growth, depending on their ecological niche, regardless of food resources [57]. The positive correlation between exposure and growth suggests that bacteria may be an important, but perhaps risky, food source for *X. laevis* tadpoles.

Risk of infection likely depends on the MHC and kinship composition of schooling tadpoles, pathogen pressure, and developmental stage. Association preferences appear to be labile in terms of MHC and kin composition within a school [42], [61]. Recent work shows that *X. laevis* tadpoles preferentially school with siblings with which they share MHC haplotypes [42]. However, among non-siblings, results differ. Tadpoles with *rr* or *gg* genotypes actively avoid non-siblings with which they share MHC alleles [61]. Tadpoles with these genotypes may avoid MHC-similar individuals to avoid reservoirs of this ubiquitous bacterial pathogen. If individuals school with others bearing the same MHC alleles as themselves, they are unlikely to be adversely affected by novel, virulent pathogens carried by these individuals, as all share similar adaptive immune systems [62]. However, should a new pathogen enter their environment, the pathogen may exploit this genetic similarity at the MHC to more quickly overwhelm the tadpoles' common immunological defenses. The cost of associating with immunologically similar individuals may be greater during susceptible periods of development, such as metamorphosis [63].

Tadpoles that developed from eggs that had been laid earlier in the evening were smaller and more likely to die than those from the same parents that had been deposited later in the evening. The ecological significance of ovum size variability in growth and survival in *X. laevis* is unclear but has been described in other amphibians in which females ‘hedge their bets’ on the environmental stability of breeding ponds [64]–[69]. Well-provisioned embryos can survive environmentally stressed conditions in which poorly provisioned eggs die. Pathogens and parasites may represent important selective pressures on the evolution of amphibian reproductive patterns [70], as well-provisioned embryos also might be better able to immunologically respond to pathogens. But these results might represent a laboratory artifact, for example, if females deposit older eggs first after being induced to oviposit.

Although our results suggest that differential susceptibility to the pathogen reflects genetic variation in resistance conferred by different MHC alleles, we did not assay pathogen load. Differences in growth and survival may have resulted from variation in tolerance of pathogen load rather than resistance to infection [71]. Although amphibian hosts typically either succumb to parasites or clear them in experimental tests [8], recent field data suggest that after experiencing an initial epizootic, surviving hosts can coexist with pathogens such as the amphibian chytrid fungus *B. dendrobatidis* [72], [73]. Whether MHC genotypes might differentially confer tolerance of pathogen load is unknown.

Despite having a comprehensive system of innate immunity that includes an extensive and exceptionally effective suite of antimicrobial peptides present in the skin [10], [74]–[76] and a well-developed adaptive immune system [10], [60], [76], whose genetics we have studied here, amphibian populations worldwide are declining as individuals succumb to pathogens [10]–[14], [17]–[22]. Antimicrobial peptides successfully inhibit the growth *in vitro* of the amphibian chytrid fungus but not *A. hydrophila* [74], [77], [78]. Both adaptive and innate immune responses may be compromised by stress, whether natural or caused by environmental perturbation, as they are regulated by the hypothalamus-pituitary-interrenal axis, which links neural, endocrine, and immune systems [57], [76]. Furthermore, survivors of mass mortality events will be subject to the compounding pressures of increased inbreeding, further loss of genetic variation, and the risk of pathogen-induced extinction if a new or recently mutated pathogen evades immune recognition in these genetically depauperate groups [9], [79]–[81].

We have presented evidence for specific MHC haplotype-based resistance to, or tolerance of, a common, if opportunistic, amphibian pathogen. Knowledge of specific resistances conferred by different genotypes may be critical to the success of captive rearing programs [13]. Moreover, the intermediate susceptibility of MHC heterozygotes to either of their potential homozygous states reinforces the importance of maintaining MHC-diverse populations if amphibians are to survive exposure to new and changing pathogens. As several pathogens have been implicated in amphibian declines, further work that examines the role of the MHC in conferring disease resistance is needed to assess the need for genetic diversity in managing amphibian conservation.
